# Comparative analysis of adenovirus, mRNA, and protein vaccines reveals context-dependent immunogenicity and efficacy

**DOI:** 10.1172/jci.insight.198069

**Published:** 2025-11-10

**Authors:** Bakare Awakoaiye, Shiyi Li, Sarah Sanchez, Tanushree Dangi, Nahid Irani, Laura Arroyo, Gabriel Arellano, Shadi Mohammadabadi, Malika Aid, Pablo Penaloza-MacMaster

**Affiliations:** 1 Department of Microbiology-Immunology, Feinberg School of Medicine, Northwestern University, Chicago, Illinois, USA.; 2 Center for Virology and Vaccine Research, Beth Israel Deaconess Medical Center (BIDMC), Boston, Massachusetts, USA.

**Keywords:** Immunology, Infectious disease, Adaptive immunity, T cells, Vaccines

## Abstract

Despite the widespread use of adenovirus, mRNA, and protein-based vaccines during the COVID-19 pandemic, their relative immunological profiles and protective efficacies remain incompletely defined. Here, we compared antigen kinetics, innate and adaptive immune responses, and protective efficacy following Ad5, mRNA, and protein vaccination in mice. Ad5 induced the most sustained antigen expression, but mRNA induced the most potent IFN responses, associated with robust antigen presentation and costimulation. Unlike Ad5 vaccines, which were hindered by preexisting vector immunity, mRNA vaccines retained efficacy after repeated use. As a single-dose regimen, Ad5 vaccines elicited higher immune responses. However, as a prime-boost regimen, and particularly in Ad5 seropositive mice, mRNA vaccines were more immunogenic than the other vaccine platforms. These findings highlight strengths of each vaccine platform and underscore the importance of host serostatus in determining optimal vaccine performance.

## Abstract 2

This study compares adenovirus, mRNA, and protein vaccines, revealing how their relative efficacies vary by platform, dosing regimen, and prior host immunity

## Introduction

The rapid development of vaccines based on different platforms, including adenoviral vector vaccines, mRNA vaccines, and protein subunit vaccines, was pivotal in curbing severe disease and death during the COVID-19 pandemic. Early in the pandemic, Ad5-based vaccines such as CanSino and Sputnik V, alongside mRNA vaccines developed by Pfizer-BioNTech and Moderna, provided efficacy against severe SARS-CoV-2 infection. Protein-based vaccines like Novavax were introduced later but also demonstrated efficacy. While each of these platforms has proven immunogenic and protective in clinical studies, their relative immunologic profiles and efficacy have not been directly compared in controlled settings.

Among COVID-19 vaccines, Ad5-based and mRNA-based vaccines emerged as leading platforms due to their potent immunogenicity. However, the influence of vaccine schedule and host serological status remains underexplored. Adenoviral vectors like Ad5 are known to elicit strong and durable T cell responses, yet their efficacy can be reduced in individuals with preexisting immunity to the vector, particularly in populations with high adenovirus seroprevalence (1). In this study, we performed a head-to-head comparison of Ad5, mRNA, and protein vaccines in mice to examine antigen expression dynamics, innate and adaptive immune responses, and protective efficacy. Our results reveal that the performance of each platform is context dependent, shaped by the vaccination schedule and the host’s serostatus. These findings provide immunological insights into 3 widely used vaccine platforms and offer guidance on tailoring vaccine strategies to specific populations.

## Results

### Antigen presentation, costimulation, and cytokine expression after vaccination with Ad5, mRNA, and protein.

**Figure 1:**
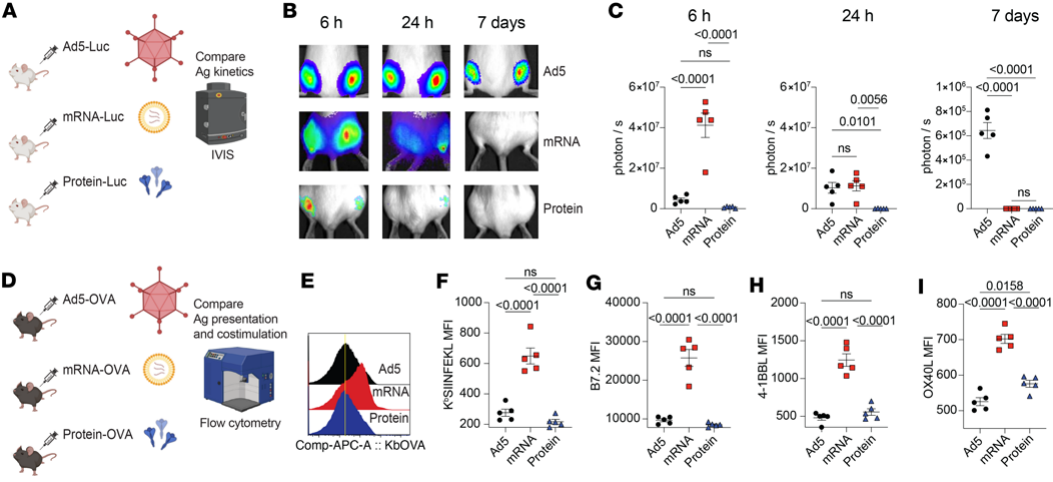
Comparison of antigen presentation and costimulation following immunization with Ad5, mRNA, and protein vaccines. (**A**) Experimental outline for comparing antigen kinetics. BALB/c mice were immunized intramuscularly with Ad5-Luc, mRNA-Luc, or Luciferase. (**B**) In vivo bioluminescence images at various time points after immunization. (**C**) Summary of antigen expression by in vivo bioluminescence. (**D**) Experimental outline for interrogating antigen presentation and costimulation at day 1 after vaccination. (**E**) Representative FACS histograms showing DC that present cognate antigen. (**F**) K^b^ SIINFEKL expression. (**G**) B7.2 expression. (**H**) 4-1BBL expression. (**I**) OX40L expression. Data are from one experiment (*n* = 5 per group). Experiments were repeated once with similar results. Indicated *P* values were calculated by ordinary 1-way ANOVA with Dunnett’s multiple comparisons. Data are shown as mean ± SEM.

There are 3 critical signals necessary for the activation of adaptive immune responses following natural infection or vaccination: (a) antigen presentation, (b) costimulation, and (c) cytokine signaling (2). We conducted experiments to compare these 3 signals after vaccination with either Ad5, mRNA, or protein (administered 1:10 with AdjuPhos). To examine antigen levels, we vaccinated BALB/c mice intramuscularly with Ad5 or mRNA expressing a luciferase reporter (Ad5-Luc or mRNA-Luc), or luciferase protein itself, and we then injected these mice with luciferin at various time points to quantify antigen levels by in vivo bioluminescence (Figure 1A). We utilized BALB/c mice because their white coat facilitates the visualization of the luciferase reporter at the site of vaccination, the quadriceps muscle. mRNA vaccination resulted in diffuse antigen expression extending beyond the injection site, while antigen expression from Ad5 and protein vaccines remained localized at a more discrete site within the quadriceps (Figure 1B). mRNA vaccination induced high antigen levels at 6 hours (h), but antigen levels rapidly declined after 24 h (Figure 1, B and C). In contrast, the Ad5 vaccine induced low antigen levels at 6 h, but antigen expression persisted longer than with the mRNA vaccine (Figure 1, B and C). With the protein vaccine, antigen levels were lower than the other vaccine platforms (Figure 1, B and C).

**Figure 2:**
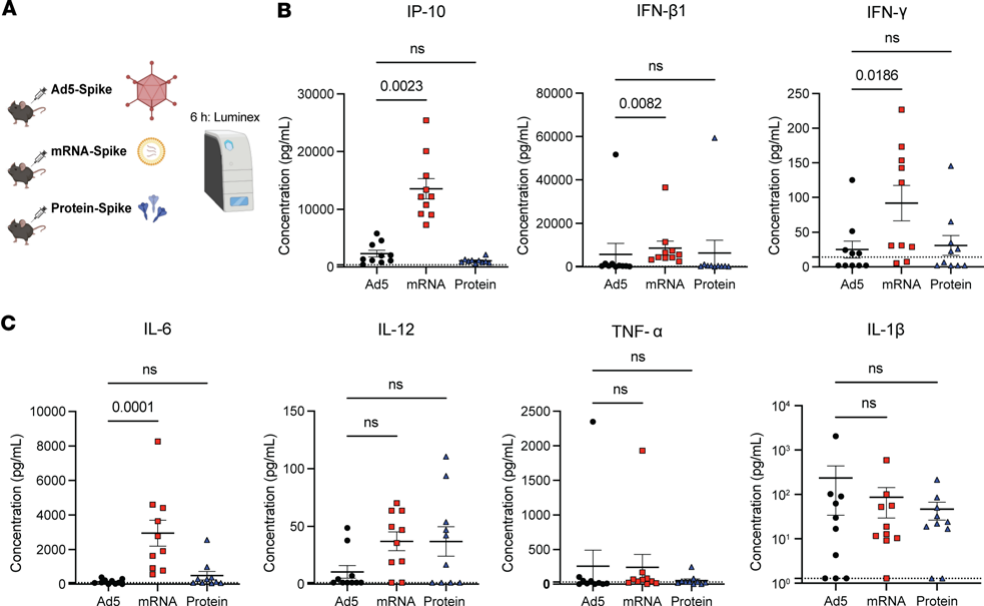
Acute cytokine profiles following immunization with Ad5, mRNA, and protein vaccines. (**A**) Experimental outline for comparing acute sera cytokine responses at 6 h after immunization in C57BL/6 mice that were immunized intramuscularly with Ad5, mRNA, and protein vaccines. (**B**) IFN-related cytokines. (**C**) Other cytokines. Data are from 2 experiments (*n* = 5 per group/experiment). Indicated *P* values were calculated by Kruskal-Wallis 1-way ANOVA test with Dunn’s multiple comparisons. Data are shown as mean ± SEM.

We then compared antigen presentation (Signal 1) by DC after immunization with the 3 vaccine platforms (Figure 1D). C57BL/6 mice were vaccinated intramuscularly with Ad5 or mRNA vaccines expressing OVA (Ad5-OVA and mRNA-OVA), or OVA protein itself. At day 3 after vaccination, we harvested draining lymph nodes and quantified antigen presentation on DCs utilizing a fluorochrome-labeled antibody recognizing MHC class I bound to SIINFEKL peptide (Figure 1D). Interestingly, mRNA vaccination generated DCs with higher levels of MHC class I bound to SIINFEKL peptide, suggesting more robust antigen presentation capacity (Figure 1, E and F). We also compared costimulation (Signal 2) by DC after immunization with the 3 vaccine platforms. mRNA vaccination induced higher expression of B7.2, 4-1BBL, and OX40L molecules on DCs, relative to the other vaccine platforms, suggesting greater costimulatory potential (Figure 1, G–I). We then compared cytokine responses (Signal 3) at 6 h after vaccination by Luminex (Figure 2A). mRNA vaccination induced higher levels of various acute cytokines, including IFN-related cytokines like γ-induced protein 10 (IP-10), IFN-β, and IFN-γ, relative to the other vaccine platforms (Figure 2B). mRNA vaccination also induced a significant increase in IL-6, but other cytokines like IL-12, TNF-α, and IL-1β were comparable across the different vaccine platforms (Figure 2C). Together, these data show differences in antigen presentation, costimulation, and acute cytokine responses between the 3 vaccine platforms.

### Single-cell gene expression analyses reveal differences in viral sensing pathways and IFN-related pathways.

**Figure 3:**
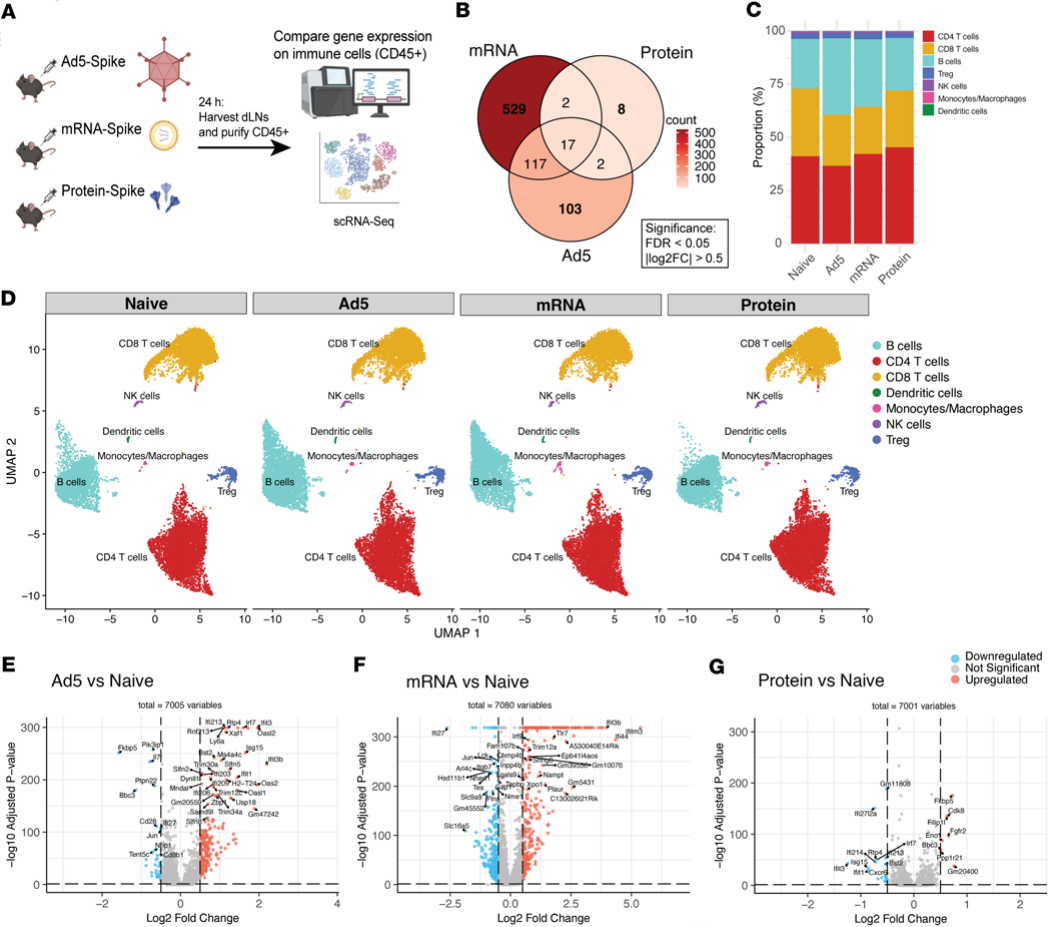
scRNA-Seq analyses reveal enrichment in IFN pathways and viral sensing pathways in mice vaccinated with mRNA. (**A**) Experimental outline for gene expression analysis of immune cells (CD45^+^) from draining lymph nodes. C57BL/6 mice were immunized intramuscularly with each respective vaccine, and at 24 h after immunization, draining lymph nodes were enriched for CD45^+^ cells using magnetic beads. (**B**) Venn diagram showing the overlap of significant differentially expressed genes (DEGs) among each vaccine group compared with naive controls. Significance was defined as FDR < 0.05 and absolute log_2_ fold change > 0.5. (**C**) Bar plot depicting the proportions of annotated cell types in each vaccination group. (**D**) UMAP visualization of single-cell transcriptomes, with cells colored and labeled by cell identity. UMAPs are shown separately for each vaccination group. (**E**–**G**) Volcano plots displaying DEGs for Ad5 versus naive, mRNA versus naive, and protein versus naive comparisons. Vertical lines indicate log_2_ fold change of 0.5; horizontal lines indicate FDR of 0.05. Genes upregulated in the vaccine group are shown in red, downregulated in blue, and nonsignificant genes in gray. Other data for this analysis are shown in Supplemental Figure 1. Data are from lymph nodes from 5 mice per group (10 lymph nodes/group).

We then performed gene expression analyses after vaccination with Ad5, mRNA, and protein. We harvested draining lymph nodes at day 1 after vaccination, followed by enrichment of live CD45^+^ cells by magnetic cell sorting and single-cell RNA-Seq (scRNA-Seq) (Figure 3A). mRNA vaccination induced the most pronounced transcriptional changes with 529 unique genes relative to naive (Figure 3B). Protein vaccination, on the other hand, induced the least transcriptional changes with only 8 unique genes relative to naive (Figure 3B and Supplemental Table 1; supplemental material available online with this article; https://doi.org/10.1172/jci.insight.198069DS1).

Most cells in the draining lymph node were lymphocytes, with a smaller proportion consisting of monocytes, macrophages, and DC (Figure 3C). Of note, Ad5 and mRNA vaccination were associated with higher frequencies of B cells, relative to naive (Figure 3D). Consistent with the Luminex data that show higher IFN responses in mRNA vaccinated mice, we observed that mRNA vaccination elicited the most potent expression of IFN-induced genes (ISGs), including those coding for Ifitm3, Ifit3, Ifi44, Ifit3, and Cxcl10 (Figure 3, E–G, and Supplemental Figure 1A). We then analyzed inflammatory pathways on specific immune cell subsets, including lymphocytes and DC. When we analyzed lymphocytes, Ad5 and mRNA vaccination were associated with enrichment in pathways involved in viral sensing, IFN-I, IFN-II, IFN-III, proteasome function, antigen processing, antigen presentation, and activation of pattern recognition receptors, relative to protein vaccination (Supplemental Figure 1, B–D). When we analyzed gene expression on DC specifically, mRNA vaccination was associated with enrichment in pathways involved in viral sensing, IFN-I, and activation of pattern recognition receptors (Supplemental Figure 1E). These data demonstrate that mRNA vaccination is associated with a more pronounced enrichment in IFN-I–induced genes, especially on DC, relative to the other vaccine platforms.

### Comparative analyses of adaptive immune responses.

**Figure 4:**
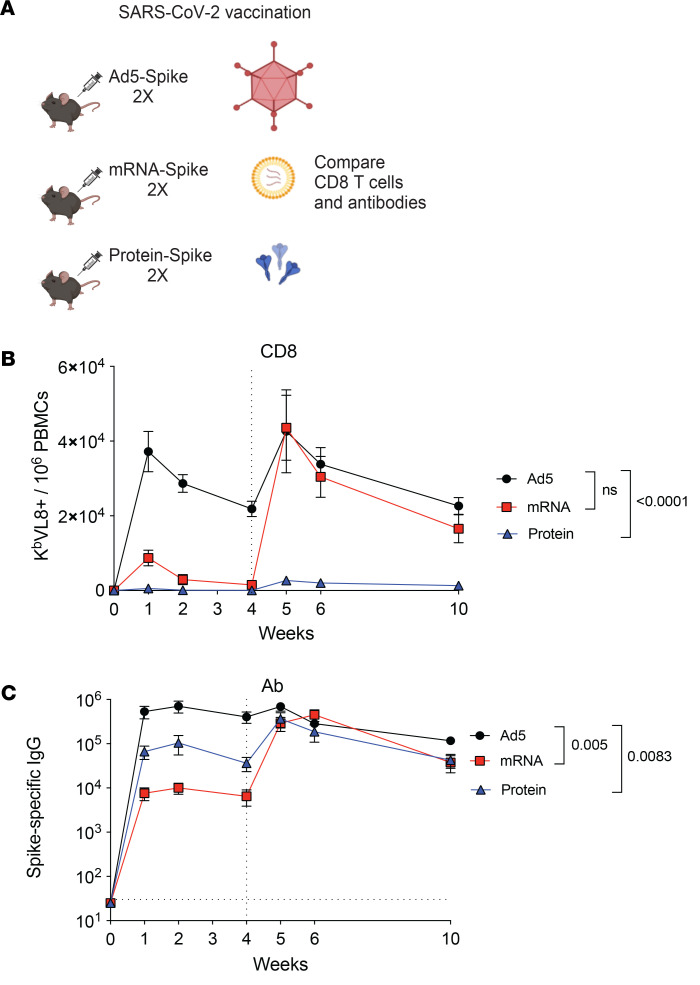
Comparative analyses of adaptive immune responses. (**A**) Experimental outline for comparing antibody and CD8 T cell responses following vaccination. C57BL/6 mice were immunized intramuscularly with each respective vaccine based on the SARS-CoV-2 spike antigen. After 4 weeks, mice were boosted homologously. (**B**) Summary of SARS-CoV-2–specific (K^b^ VL8^+^) CD8^+^ T cells in PBMCs. (**C**) Summary of SARS-CoV-2–specific antibody titers in sera. Data from 4 experiments (*n* = 5 mice per group/experiment). The vertical dashed line indicates the time of boosting. Indicated *P* values were calculated by ordinary 1-way ANOVA with Dunnett’s multiple comparisons at week 10. Data are shown as mean ± SEM.

We then compared T cell and antibody responses after vaccination. C57BL/6 mice were vaccinated intramuscularly, with Ad5 or mRNA vaccines expressing the SARS-CoV-2 spike protein (Ad5-spike and mRNA-spike), or SARS-CoV-2 spike protein itself, and analyzed CD8 T cell responses by tetramer stains and antibody responses by ELISA (Figure 4A). After a single prime, Ad5 elicited the most potent CD8 T cell response (Figure 4B). However, following a booster vaccination, CD8 T cell responses were comparable among the Ad5 and mRNA groups. Protein vaccination elicited low CD8 T cell responses that were near the limit of detection. We also compared antibody responses between the different vaccine platforms and observed greater responses with Ad5 vaccines, compared with the other vaccines (Figure 4C). Overall, these initial data show that Ad5 is more immunogenic than the other vaccines when comparing single-dose regimens.

**Figure 5:**
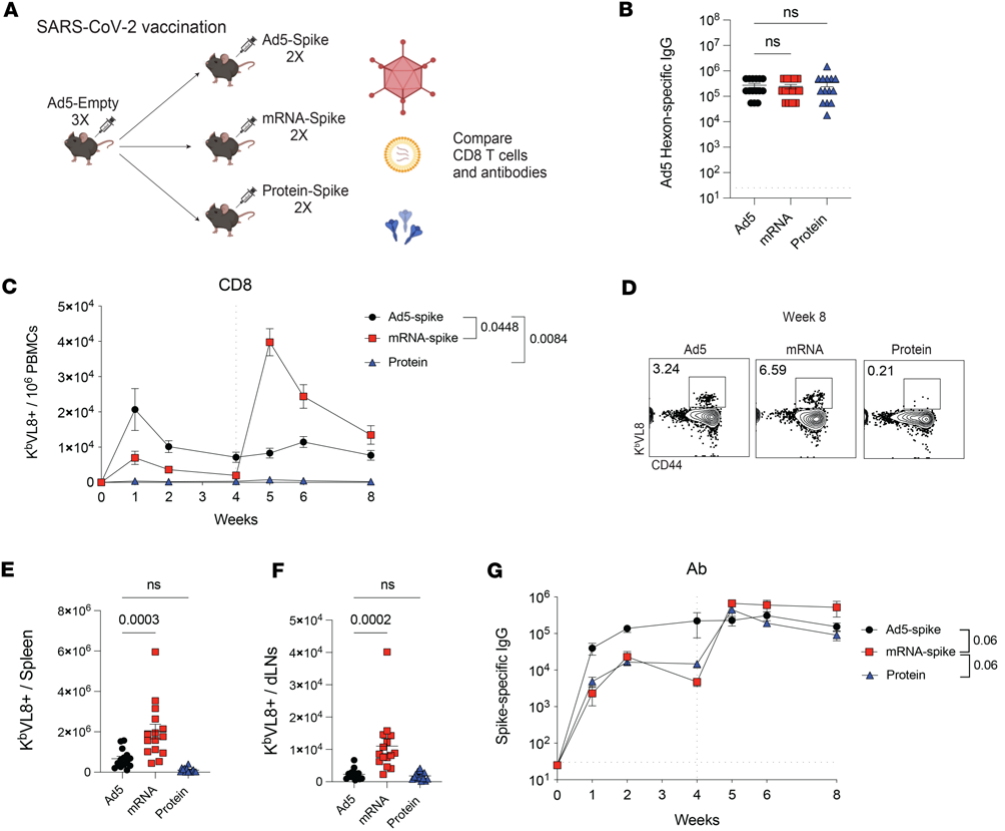
Comparative analyses of adaptive immune responses in Ad5 seropositive hosts. (**A**) Experimental outline for comparing vaccine-elicited responses. First, mice were made Ad5 seropositive by injecting them with 1 × 10^9^ PFUs of Ad5-Empty per mouse, once every 3 weeks for a total of 3 doses. These mice were then immunized intramuscularly with each respective vaccine based on the SARS-CoV-2 spike antigen. After 4 weeks, mice were boosted homologously. (**B**) Ad5 seropositivity was confirmed by Ad5 hexon-specific antibody titers. (**C**) Summary of SARS-CoV-2–specific (K^b^ VL8^+^) CD8^+^ T cells in PBMCs. (**D**) Representative FACS plots showing SARS-CoV-2–specific CD8^+^ T cell in PBMCs at week 8. (**E**) Summary of SARS-CoV-2–specific CD8^+^ T cells in spleen. (**F**) Summary of SARS-CoV-2–specific CD8^+^ T cells in draining lymph nodes. (**G**) Summary of SARS-CoV-2–specific antibody titers in sera. Data from 4 experiments (*n* = 5 mice per group/experiment). The vertical dashed line indicates the time of boosting. Indicated *P* values in **C**, **E**, and **F** were calculated by ordinary 1-way ANOVA with Dunnett’s multiple comparisons (*P* value for **C** is from week 8). Indicated *P* values in **F** were calculated by 2-way ANOVA with Holm-Šídák multiple-comparison test (*P* value for **F** is from week 8). Data are shown as mean ± SEM.

Ad5 is highly seroprevalent in humans (1). Given the widespread seroprevalence of Ad5, we reasoned that a more appropriate comparison of vaccine platforms would need to account for the high level of Ad5 seropositivity in the human population. To model this, we first rendered mice seropositive to Ad5 by immunizing them 3 times with an Ad5 vector devoid of any vaccine antigen (Ad5-Empty) (Figure 5A). This regimen generated high titers of Ad5-specific antibodies above > 1 × 10^5^ endpoint titer, recapitulating the Ad5 seropositivity observed in humans (Figure 5B). These Ad5-seropositive mice were then immunized with vaccines based on Ad5, mRNA, or protein, and adaptive immune responses were evaluated. In Ad5-seropositive mice, a prime-boost regimen with mRNA elicited more robust CD8 T cell responses than the other vaccine regimens (Figure 5, C–F). Moreover, CD8 T cells induced by the mRNA regimen showed higher coexpression of cytokines such as IFN-γ, TNF-α, and IL-2 (Supplemental Figure 2, A–C). We did not observe statistically significant differences in CD4 T cell responses between the different vaccine platforms (Supplemental Figure 2D).

We also compared CD8 T cell subset differentiation, and we showed that most antigen-specific CD8 T cells generated by Ad5 exhibited a short-lived effector cell (SLEC) phenotype, whereas most antigen-specific CD8 T cells generated by mRNA and protein showed a memory precursor effector cell (MPEC) phenotype (Supplemental Figure 3, A and B). There was also a pattern of improved antibody responses after mRNA vaccination, but the difference was not statistically significant (*P* = 0.06) (Figure 5G). Overall, these data demonstrate that, in an Ad5 seropositive host, a prime-boost regimen with mRNA is more immunogenic than the other vaccine platforms in terms of CD8 T cells responses, whereas CD4 T cells and antibodies are not substantially different.

### Effects of preexisting immunity.

As previously discussed, a major limitation of utilizing Ad5 vectors is their high seroprevalence, which can limit their clinical efficacy. In an earlier study, we demonstrated that, during Ad5 immunization, preexisting Ad5-specific antibodies can accelerate the clearance of the Ad5 vector at the site of immunization, limiting antigen expression by the vector (3). We also observed that prior immunization of mice with an Ad5-Empty vector limits the immunogenicity of the Ad5 vector platform after subsequent immunizations (Supplemental Figure 4, A–C), consistent with prior studies (4). These negative effects of preexisting immunity have been the basis for developing adenovirus vector platforms with lower seroprevalence, including Ad26 (5, 6). Besides using a different adenovirus serotype for booster vaccination, heterologous Ad/mRNA vaccination regimens have shown benefits over homologous vaccination regimens (7). We reasoned that boosting with a protein vaccine could also help overcome preexisting immunity in Ad5-primed mice, potentiating adaptive immune responses. Consistent with our hypothesis, we show that a heterologous Ad5/protein regimen can overcome some of the limitations of the homologous Ad5/Ad5 regimen, resulting in more potent humoral responses (Supplemental Figure 4, D–I).

So far, we have shown that prior immunization with Ad5 hampers the reutilization of this vector, consistent with prior reports. But it is unclear whether the same occurs with mRNA vaccines. Prior studies have shown that mRNA vaccines elicit antibodies against the lipid nanoparticle (LNP) carrier (8, 9), but whether these hamper mRNA vaccines remain unknown. To examine whether prior immunization with mRNA vaccines hampers the reutilization of mRNA vaccines, we immunized mice 3 times with an irrelevant mRNA vaccine encoding the SARS-CoV-2 spike protein or PBS control. After several weeks, these mice were immunized with an mRNA vaccine encoding a different antigen, the lymphocytic choriomeningitis virus (LCMV) glycoprotein (mRNA-LCMV) (Supplemental Figure 5A). Interestingly, mice that were previously immunized with the mRNA-spike vaccine generated similar LCMV-specific immune responses, relative to control mice (Supplemental Figure 5, B–D). We recapitulated these findings in another model, showing that prior immunization with an mRNA-OVA vaccine did not hamper immune responses to a different mRNA vaccine encoding the mouse hepatitis virus spike protein (mRNA-MHV) (Supplemental Figure 5, E–G). Collectively, these data suggest that, in contrast to Ad5 vaccines, prior immunization with mRNA vaccines does not impair the reutilization of mRNA vaccines.

### Comparative efficacy of Ad5, mRNA, and protein vaccines following stringent pathogen challenge.

**Figure 6:**
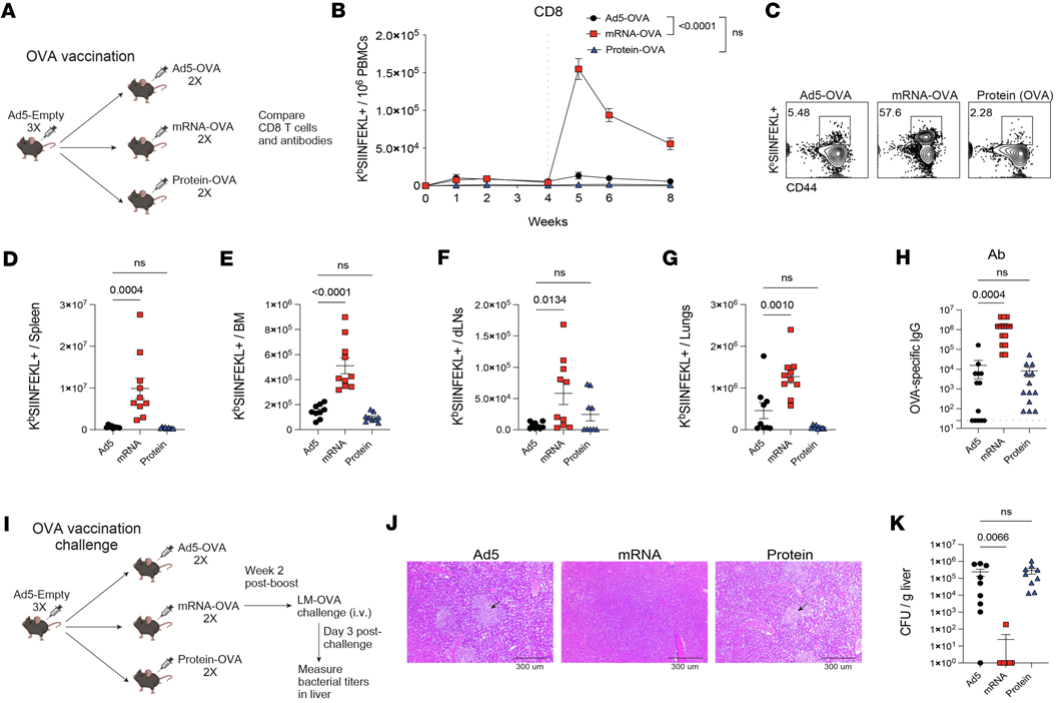
Protective efficacy following vaccination. (**A**) Experimental outline for comparing vaccine-elicited responses and immune protection. First, mice were made Ad5 seropositive by injecting them with 1 × 10^9^ PFUs of Ad5-Empty per mouse, once every 3 weeks for a total of 3 doses. These mice were then immunized intramuscularly with each respective vaccine based on the OVA antigen. After 4 weeks, mice were boosted homologously. (**B**) Summary of OVA-specific (K^b^ SIINFEKL^+^) CD8^+^ T cells in PBMCs. (**C**) Representative FACS plots showing OVA-specific CD8^+^ T cell in PBMCs. (**D**–**G**) Summary of OVA-specific CD8^+^ T cells in spleen (**D**), bone marrow (**E**), draining lymph nodes (**F**), and lungs (**G**). (**H**) OVA-specific antibody titers in sera. Data from **C**–**H** are from week ~8. (**I**) Experimental outline for comparing protective efficacy. (**J** and **K**) On day 3 after LM-OVA challenge, livers were harvested for H&E-stains (**J**) and measuring bacterial loads (**K**). Scale bars: 300 μm. Black arrows in **J** denote areas of necrosis. Data from 2 experiments (*n* = 4–5 mice per group/experiment). The vertical dashed line indicates the time of boosting. For **B** and **D**–**H**, indicated *P* values were determined by ordinary 1-way ANOVA test with Dunnett’s multiple comparisons (*P* values for **B** and **H** are from week 8). For **K**, indicated *P* values were determined by Kruskal-Wallis test with Dunn’s multiple-comparison test. Data are shown as mean ± SEM.

In the prior sections, we have compared immune responses following Ad5, mRNA, and protein vaccination. We did not compare vaccine efficacy using SARS-CoV-2 challenges, because SARS-CoV-2 is rapidly cleared in vaccinated animals, not allowing us to detect differences in viral control between vaccinated groups. Even a single dose with SARS-CoV-2 vaccines confers robust protection in animal models, making it difficult to ascertain differences in immune protection among various vaccine regimens (10–15). To detect differences in immune protection, we relied on a more stringent challenge model based on Listeria monocytogenes. First, Ad5-seropositive mice were vaccinated with Ad5-OVA, mRNA-OVA, or OVA protein, followed by immunogenicity and efficacy studies using a stringent Listeria challenge model (Figure 6A). Consistent with our prior results, the mRNA vaccine regimen elicited greater OVA-specific CD8 T cell responses in blood and tissues, compared with the other vaccine platforms (Figure 6, B–G). There were also improved antibody responses in mRNA-vaccinated mice, relative to the other vaccine regimens (Figure 6H). Similar to our studies using the spike antigen model, we did not observe differences in CD4 T cell responses specific for OVA (Supplemental Figure 6, A and B). Moreover, we did not observe differences in CD4^+^FoxP3^+^ Tregs, suggesting that the differences in vaccine responses were not due to this regulatory cell subset (Supplemental Figure 6, C and D).

To compare vaccine efficacy, we challenged mice i.v. with a supra-lethal dose (1 × 10^7^ CFU/mouse) of Listeria monocytogenes expressing OVA (LM-OVA) (Figure 6I). This lethal challenge model is particularly stringent, as most experimental Listeria vaccines are unable to control systemic bacterial dissemination (16), rendering it well suited to compare differences in vaccine efficacy. As expected, mice vaccinated with Ad5 or protein exhibited disseminated necrosis and high bacterial titers in the liver after an LM-OVA challenge (Figure 6, J and K). In contrast, most mice vaccinated with mRNA showed sterilizing immunity, with no evidence of Listeria infection (Figure 6, J and K). These data suggest that mRNA vaccines are more effective than Ad5 vaccines and protein vaccines, especially after a prime-boost regimen and in an Ad5 seropositive host.

## Discussion

This study provides a side-by-side comparison of Ad5, mRNA, and protein vaccines across multiple immunological parameters and in different serological contexts. While previous studies, including those comparing mRNA, Ad26, and protein platforms in human volunteers, have offered important insights (17), their interpretation has been confounded by host variability, including wide genetic diversity and uncontrolled preexisting immunity to adenoviruses. In contrast, our study leverages a controlled murine model to systematically assess the immunological differences between vaccine platforms, enabling clear attribution of observed effects to the vaccines themselves rather than to host background, immune histories, or genetic variation. By incorporating both naive and Ad5-seropositive mice, we further model clinically relevant contexts that may influence vaccine efficacy.

We show that, while Ad5 elicits robust immunity after a single dose in naive hosts, its relative performance declines after readministration. In contrast, mRNA vaccines induce modest immune responses after a single dose but robust immune responses after a booster dose. These findings underscore that vaccine performance is context dependent, shaped not only by each platform’s immunobiology but also by the number of shots administered and the host’s serological status. It is important to note that the immunogenicity of mRNA vaccines is not entirely impervious to preexisting immunity. In a previous study, we show that antibody responses against the encoded antigen can accelerate clearance of this cognate antigen at the immunization site, affecting the priming of immune responses (18).

Our studies do not necessarily conclude that adenovirus vaccines are less effective than mRNA vaccines. There are advantages of adenoviral vaccines over mRNA vaccines, including their low cost and stability, which makes them better suited for low-income countries, as well as their potent immunogenicity, immune durability, and protective efficacy after just a single dose (19). Adenovirus vectors also induce more durable antigen expression lasting more than a week, potentially making them more appropriate for applications that require long-term protein expression. mRNA vaccines, on the other hand, induce an acute “burst” in antigen expression at significantly higher levels than the other vaccines as measured by in vivo bioluminescence, which could make them more appropriate for applications that require transient antigen expression. These distinct antigen kinetics likely contribute to the differences in immune responses between the vaccines. The “burst” in antigen expression observed with mRNA vaccines is associated with more potent DC activation, including higher MHC class I and costimulatory molecule expression relative to the other vaccines, which may contribute to the high anamnestic capacity of memory CD8 T cells in mRNA-immunized mice. Additionally, mRNA vaccines induce higher levels of inflammatory cytokines such as IP-10, IFN-β, and IFN-γ, consistent with a robust interferon signature, which was validated at the gene expression level and the protein level. Prior studies have shown that IL-6 is critical for the adjuvant activity of LNPs (20), and consistent with this, we observed significantly higher IL-6 levels following mRNA vaccination compared with the other vaccine platforms, suggesting that IL-6 plays a key role in shaping immune responses to mRNA vaccines.

The protective efficacy of the 3 vaccines was then compared using a stringent Listeria challenge model. This challenge model showed that an mRNA prime-boost regimen confers significantly higher protection than the other vaccine regimens. This high level of immune protection against Listeria in mRNA vaccinated mice could be explained mostly by memory CD8 T cells, given that these cells play a critical role in the clearance of intracellular infections.

Our study has limitations. First, our bioluminescence experiments rely on a luciferase reporter to quantify antigen expression. Lack of bioluminescence does not necessarily indicate complete antigen clearance but rather the loss of luciferase’s enzymatic activity. Antigen fragments may persist beyond the detection window and continue priming immune responses. Second, we did not assess the effects of additional booster doses beyond a prime-boost regimen. Given that many individuals have already received multiple boosters during the COVID-19 pandemic, it would be valuable to investigate how repeated boosters influence immune responses across different vaccine platforms. Overall, our findings demonstrate that the relative efficacy of different vaccine platforms is context dependent. After a single dose, Ad5 vaccines induce long-term antigen expression associated with durable immune responses, making them well suited as single-dose vaccination strategies. While mRNA vaccines induce modest primary CD8 T cell responses after a single dose, these responses become highly anamnestic upon boosting immunization. mRNA vaccines are also not affected by antivector immunity, enabling repeated use of this vaccine platform. These data highlight the importance of considering population serological background and context when selecting vaccines against novel diseases.

## Methods

### Mice and immunizations

#### Sex as a biological variable.

Our study examined male and female animals, and similar findings are reported for both sexes. Six- to 8-week-old C57BL/6 mice were used. Mice were immunized intramuscularly with each respective vaccine. Mice were purchased from Jackson laboratories (approximately half males and half females) and were housed at Northwestern University’s Center for Comparative Medicine (CCM).

### Reagents, flow cytometry, and equipment

Dead cells were gated out using Live/Dead fixable dead cell stain (Invitrogen). SARS-CoV-2 spike peptide pools were used for intracellular cytokine staining (ICS) and these were obtained from BEI Resources. Biotinylated MHC class I monomers were used for detecting antigen-specific CD8 T cells and were obtained from the NIH tetramer facility at Emory University (Atlanta, Georgia, USA.). MHC class II tetramers were used for detecting antigen-specific CD4 T cells and were also obtained from the NIH tetramer facility at Emory University. Cells from PBMCs and tissues were stained with fluorescently labeled antibodies against CD8α (53-6.7 on PerCP-Cy5.5), CD44 (IM7 on FITC), CD127 (A7R34 on Pacific Blue), KLRG-1 (2F1 on PE-Cyanine7), IL-2 (JES6-5H4 on PE), TNF-α (MP6-XT22 on PE-Cy7), IFN-γ (XMG1.2 on APC), or tetramers (APC or PE). All fluorescently labeled antibodies were purchased from BD Pharmingen, except for anti-CD127 and anti-CD44 (which were purchased from BioLegend). Flow cytometry samples were acquired with a Becton Dickinson Canto II or an LSRII and analyzed using FlowJo v10 (Treestar).

### SARS-CoV-2 spike, OVA, and Ad5 hexon-specific ELISA

Binding antibody titers were measured using ELISA as described previously (15, 16, 18, 21, 22). In brief, 96-well flat bottom plates MaxiSorp (Thermo Scientific) were coated with 0.1 μg/well of the respective protein, for 48 h at 4°C. Plates were washed with PBS + 0.05% Tween-20. Blocking was performed for 4 h at room temperature with 200 μL of PBS + 0.05% Tween-20 + bovine serum albumin. In total, 6 μL of sera were added to 144 μL of blocking solution in the first column of the plate, 1:3 serial dilutions were performed until row 12 for each sample, and plates were incubated for 60 minutes at room temperature. Plates were washed 3 times followed by the addition of goat anti-mouse IgG horseradish peroxidase-conjugated (Southern Biotech) diluted in blocking solution (1:5,000), at 100 μL/well and incubated for 60 minutes at room temperature. Plates were washed 3 times and 100 μL /well of Sure Blue substrate (Sera Care) was added for approximately 8 minutes. The reaction was stopped using 100 μL/well of KPL TMB stop solution (Sera Care). Absorbance was measured at 450 nm using a Spectramax Plus 384 (Molecular Devices). SARS-CoV-2 spike protein was produced in-house using a plasmid produced under HHSN272201400008C and obtained from BEI Resources, NIAID, NIH: vector pCAGGS containing the SARS-related coronavirus 2; Wuhan-Hu-1 spike glycoprotein gene (soluble, stabilized). OVA protein was purchased from Worthington Biochemical (catalog LS003049). Ad5 hexon protein was purchased from BioRad (catalog MPP002).

### Ad5, mRNA, and protein vaccines

Ad5 (1 × 10^8^ VP), mRNA (5 μg), and protein (10 μg) vaccines were diluted in PBS and administered intramuscularly (50 μL/quadriceps). These doses are standard for each vaccine and were selected based on prior dose-escalation studies by us and others, showing that they elicit maximal immune responses (immune responses plateau at the doses tested and are not significantly improved with higher doses) (6, 23, 24). Protein vaccines were formulated 1:10 in AdjuPhos. Ad5 vaccines were purchased from the Iowa Vector Core. SARS-CoV-2 spike protein vaccine was produced in house using a plasmid produced under HHSN272201400008C and obtained from BEI Resources, NIAID, NIH: vector pCAGGS containing the SARS-related coronavirus 2; Wuhan-Hu-1 spike glycoprotein gene (soluble, stabilized). OVA protein vaccine was purchased from Worthington Biochemical (catalog LS003049).

We synthesized mRNA vaccines in house. mRNA vaccines encoding for the codon-optimized proteins. Constructs were purchased from Integrated DNA Technologies (IDT) or Genscript, and contained a T7 promoter site for in vitro transcription of mRNA. The sequences of the 5′- and 3′-UTRs were identical to those used in a previous publication (15). All mRNAs were encapsulated into LNPs using the NanoAssemblr Benchtop system (Precision NanoSystems) and confirmed to have similar encapsulation efficiency (~95%). mRNA was diluted in Formulation Buffer (catalog NWW0043, Precision NanoSystems) to 0.17 mg/mL and then run through a laminar flow cartridge with GenVoy ILM encapsulation lipids (catalog NWW0041, Precision NanoSystems) with N/P (Lipid mix/mRNA ratio of 4) at a flow ratio of 3:1 (RNA: GenVoy-ILM), with a total flow rate of 12 mL/min, to produce mRNA–LNPs (mRNA-LNPs). mRNA-LNPs were evaluated for encapsulation efficiency and mRNA concentration using RiboGreen assay using the Quant-iT RiboGreen RNA Assay Kit (catalog R11490, Invitrogen, Thermo Fisher Scientific).

### scRNA-Seq data acquisition

C57BL/6 mice were immunized intramuscularly with each respective vaccine. At day 1 post-vaccination, lymph nodes from 5 mice (10 lymph nodes/group) were pooled and MACS-sorted with a CD45 MACS positive selection kit (STEMCELL) and used for single-cell sequencing. The sorted cells were used for single-cell sequencing, and libraries were generated using 10x Genomics 3′ kits. scRNA-Seq data from mouse samples were processed using the Cell Ranger (cloud analysis) pipeline developed by 10x Genomics. Raw base call (BCL) files were demultiplexed into FASTQ files using the cellranger mkfastq command, with sample sheet information specifying index assignment. If FASTQ files were already available, this step was skipped. Reads were aligned to the mouse reference genome (mm10) using the cellranger count pipeline with default parameters. The mm10 reference transcriptome provided by 10x Genomics (refdata-gex-mm10-2020-A) was used for alignment and gene annotation. During this process, Cell Ranger performed read alignment using STAR, filtered and corrected barcodes, quantified unique molecular identifiers (UMIs), and generated a gene-cell count matrix. The resulting filtered_feature_bc_matrix directory containing the gene-barcode count matrix was used for downstream analysis. Cell Ranger also provided quality control metrics and summary statistics in the web_summary.html file. scRNA-Seq was performed at the Center for Virology and Vaccine Research at BIDMC.

### scRNA-Seq data analysis

Gene expression analysis was performed using R version 4.5.0, Bioconductor version 3.21, and Seurat version 5.3.0. Cells were filtered to retain those expressing between 300 and 5,000 genes, with fewer than 30,000 total UMI counts, and less than 5% mitochondrial gene content. Genes detected in at least 3 cells were retained for downstream analysis. Data normalization was performed using the LogNormalize method in Seurat, and 2,000 highly variable genes were selected using the ‘vst’ method. Doublet cells were identified and removed using the scDblFinder package (v1.10.0) with default parameters. Following normalization and scaling, principal component analysis (PCA) was performed, and samples were integrated using Seurat’s IntegrateLayers function with canonical correlation analysis (CCA) integration. Clustering was conducted using 25 principal components for both FindNeighbors and RunUMAP, and clusters were identified with a resolution of 3 using FindClusters. Cell types were annotated using the R package SingleR version 2.10.0 with the Immunological Genome Project (ImmGen) reference and refined manually based on canonical marker gene expression. The presence of Itgax (Cd11c) in clusters was used for DC annotation. For downstream analyses, cell types of interest were subset and renormalized using the same workflow.

Differential gene expression (DEG) analysis was performed using Seurat’s FindMarkers function (Wilcoxon test), comparing each vaccine group to the naive group, with a minimum detection threshold of 10% of cells (min.pct = 0.1). Ribosomal and mitochondrial genes were excluded from DEG results prior to downstream analysis. Genes with an absolute log_2_ fold change greater than 0.5 and a Benjamini-Hochberg adjusted FDR less than 0.05 were considered significant.

For pathway enrichment analysis, the fgsea package (v1.34.0) was used. Differentially expressed genes for each cell type were identified using a minimum detection threshold of 5% of cells (min.pct = 0.05). Genes were then ranked for each comparison using the metric sign(log_2_FC) × –log_10_(*P* value). Gene sets included the Hallmark and C2 curated collections from MSigDB (v2023.1), the Blood Transcriptome Modules (BTM), and in-house curated gene sets. Pathways with FDR-adjusted p-values less than 0.05 were considered significantly enriched. Figures were generated using Seurat and ggplot2 (v3.5.2). Volcano plots were created using EnhancedVolcano (v1.27.0), and Venn diagrams were generated with ggVennDiagram (v1.5.2). Refer to the following papers for additional information: Seurat (25–29); scDblFinder (30); MSigDB and GSEA (31); and R and Bioconductor (32).

### Multiplex cytokine/chemokine assay

Blood samples were centrifugated at 21,130*g* for 10 min at 4°C to separate the serum. The serum samples were collected and frozen in –80°C until its use. A multiplex cytokines/chemokines kit was purchased from Mesoscale Diagnostics LLC or Sigma Aldrich (Luminex) and used for quantifying serum cytokines/chemokines.

### Listeria quantification

Spleens were collected from infected mice on day 3 after challenge. Bacterial titers were quantified by homogenizing tissues through a 70 μm strainer and resuspended in 1% Triton. Ten-fold serial dilutions were created in 1% Triton and added dropwise onto 6-well BHK agar plates. Plates were manually rocked and then incubated at 37°C 5% CO_2_ for 48 h. Colonies were counted the next day.

### In vivo bioluminescence

After vaccinating mice with Ad5 or mRNA expressing a luciferase reporter (Ad5-Luc or mRNA-Luc), or luciferase protein itself, luciferin (GoldBio, catalog LUCK-100) was administered intraperitoneally 15 min before imaging to quantify luciferase expression, as done previously (3, 18). Mice were anesthetized and imaged using a SII Lago IVIS Imager (Spectral Instruments Imaging). Region of interest (ROI) bioluminescence was used to quantify signal.

### Statistics

Statistical analysis were performed using 1-way or 2-way ANOVA with appropriate multiple-comparison tests, or nonparametric tests as indicated in figure legends. Horizontal dashed lines in data of figures represent the limit of detection. Vertical dashed lines indicate time of boosting immunizations. Data were analyzed using Prism version 10 (Graphpad). A *P* value less than 0.05 was considered significant.

### Study approval

#### Mouse studies.

All mouse experiments were performed with approval from the IACUC of Northwestern University (study approval nos. IS00003258, IS00015002, and IS00008785). All mouse experiments with BL2 agents were performed with approval from the IACUC of Northwestern University.

### Data availability

scRNA-Seq data were deposited in the NCBI Gene Expression Omnibus (GEO) database under accession number GSE302672, at https://www.ncbi.nlm.nih.gov/geo/query/acc.cgi?acc=GSE302672 Other data are available upon request. Raw data associated with the main article and supplemental material are included in the Supporting Data Values file.

## Author contributions

PPM, BA, and SS designed the experiments. BA, SL, SS, TD, NI, GA, and LA performed the experiments. MA and SM analyzed the gene expression data. PPM and BA wrote the manuscript with feedback from all authors.

## Funding support

Research reported in this publication was supported by Northwestern University and BIDMC.

## Supplementary Material

Supplemental data

Supplemental table 1

Supporting data values

## Version 1 11/10/2025

Electronic publication
