# Supplementary material for: Comparative analysis of adenovirus, mRNA, and protein vaccines reveals context-dependent immunogenicity and efficacy
Source: JCI Insight. 2025 Nov 10;10(21):e198069. doi: 10.1172/jci.insight.198069 (PMC12643482; doi:10.1172/jci.insight.198069)
Supplement: Supplemental data [file jciinsight-10-198069-s161.pdf]

Comparative analysis of adenovirus, mRNA, and protein vaccines reveals context-dependent immunogenicity and efficacy

Bakare Awakoaiye[1], Shiyi Li[1], Sarah Sanchez[1], Tanushree Dangi[1], Nahid Irani[1], Laura Arroyo[1], Gabriel Arellano[1], Shadi Mohammadabadi[2], Malika Aid[2], Pablo Penaloza-MacMaster*[1, 2]

[1]Department of Microbiology-Immunology, Feinberg School of Medicine, Northwestern University, Chicago, IL 60611, USA. [2]Center for Virology and Vaccine Research, Beth Israel Deaconess Medical Center, Harvard Medical School, Boston, MA 02115, USA.

***Correspondence:** Pablo Penaloza-MacMaster (ppenaloz@bidmc.harvard.edu)

Figure S1

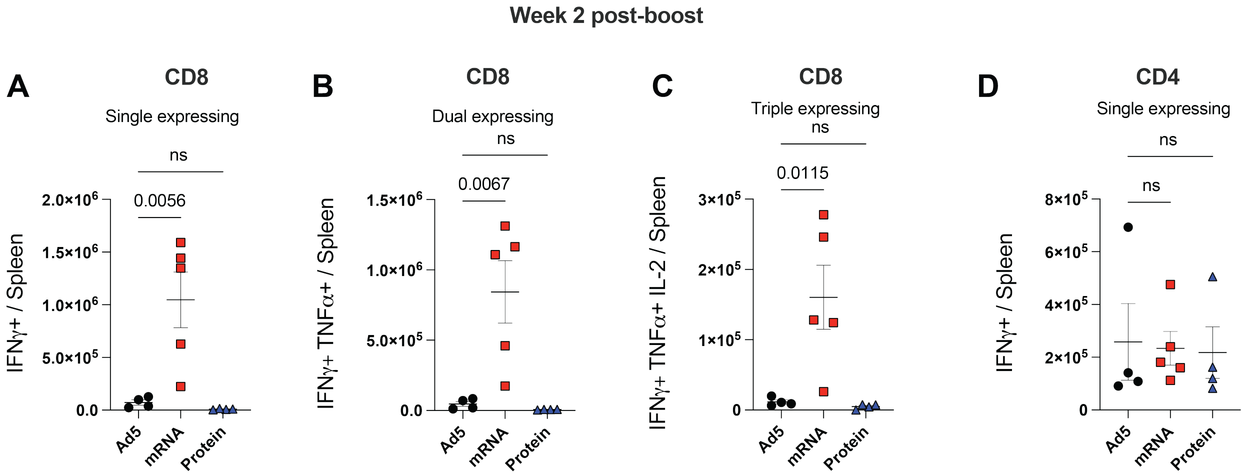

Supplemental Figure 1. Cytokine expression by virus-specific T cells following immunization with Ad5, mRNA, and protein vaccines. Experimental outline was similar to that in Figure 6A. Numbers of spike-specific CD8 T cells that express IFN-γ (**A**); IFN-γ and TNF-α (**B**); and IFN-γ, TNF- IL-2 (**C**) in spleen at week 2 post-boost. (**D**) Numbers of spike-specific CD4 T cells that express IFN-γ in spleen at week 2 post-boost. Spike peptide pool stimulations were performed for 5 h. Data from one experiment (n=5 mice per group). Experiment was repeated once with similar results. Indicated *P* values were calculated by ordinary one-way ANOVA with Dunnett's multiple comparisons. Error bars represent SEM.

Figure S2

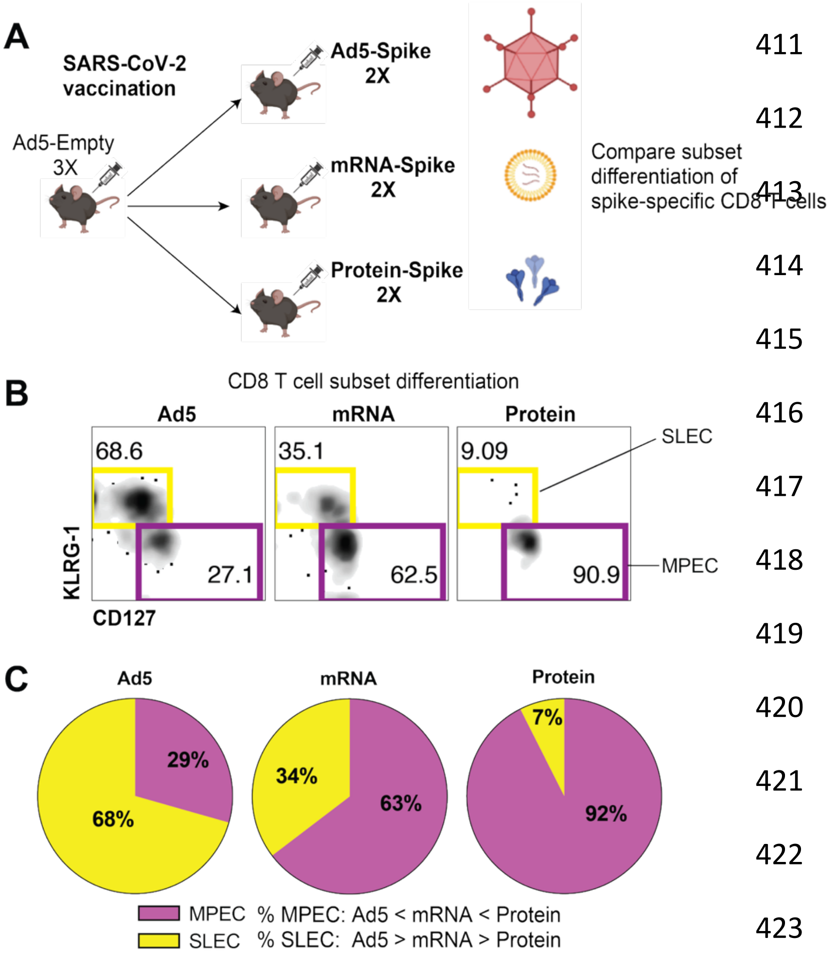

Supplemental Figure 2. Comparative analyses of CD8 T cell subset differentiation. (**A**) Experimental outline was similar to that in Figure 6A. (**B**) Representative FACS plots of short-lived effector cells (SLECs) and memory precursor effector cells (MPECs) populations, gated on SARS-CoV-2–specific (K^b VL8+) CD8 T cells (**C**) Pie diagrams showing CD8 T cell subsets. Data from one experiment (n=4-5 mice per group). Experiment was repeated once with similar results.

Figure S3

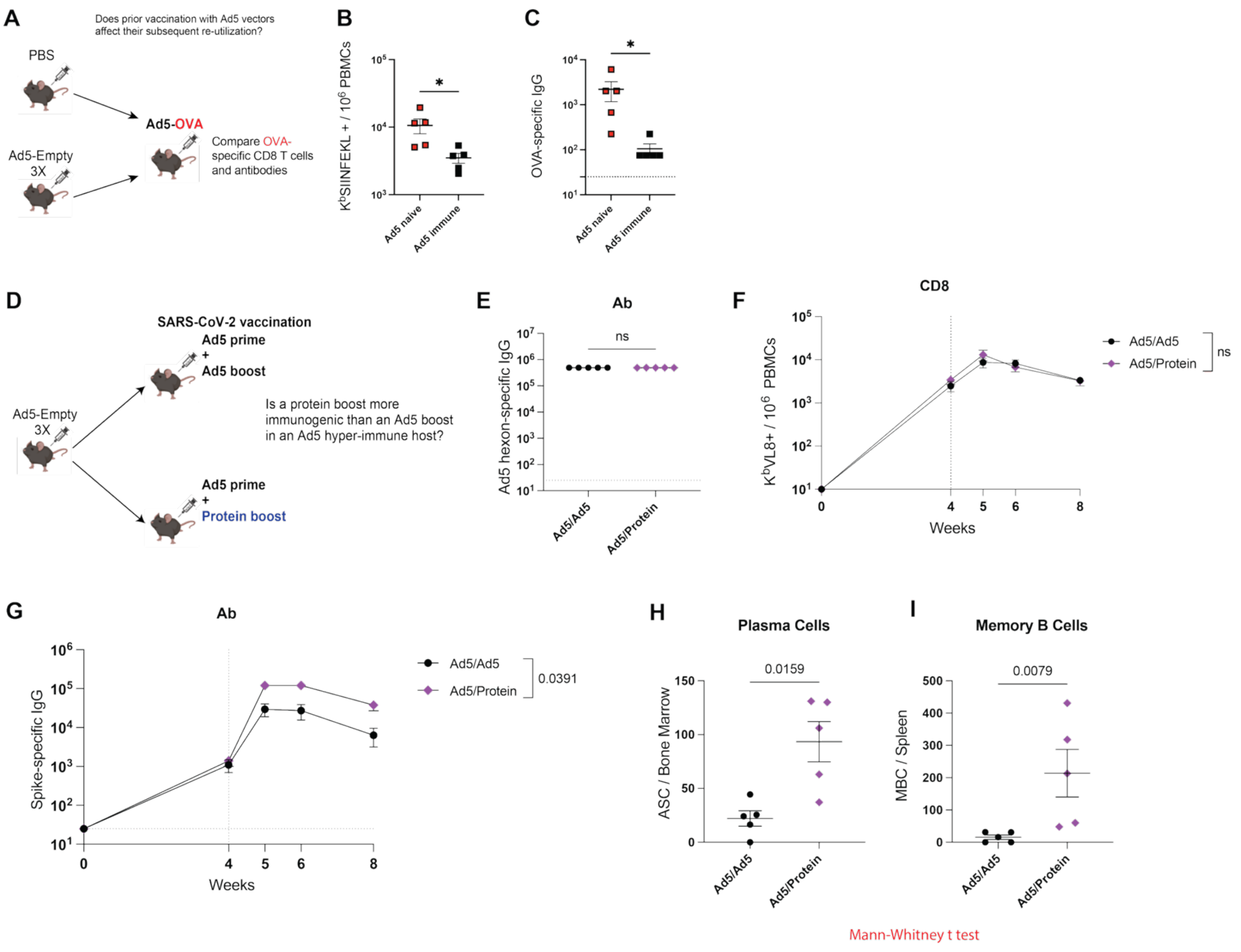

Supplemental Figure 3. Prior immunization with Ad5 renders Ad5 vectors less immunogenic. (**A**) Experimental outline for comparing Ad5 vaccine-elicited responses in mice that had been previously immunized with Ad5 vectors. First, Ad5 seropositivity was induced by injecting C57BL/6 mice with Ad5-Empty, injected intramuscularly, once every 3 weeks for a total of three doses. Control mice were injected with PBS. Mice were then immunized with Ad5-OVA and immune responses were measured at week 2 post-immunization. (**B**) Summary of OVA-specific (K^b SIINFEKL+) $CD8^+$ T cells in PBMCs. (**C**) Summary of OVA-specific antibody titers in sera. (**D**) Experimental outline for determining whether a heterologous Ad5/protein regimen elicits superior immune responses, relative to a homologous Ad5/Ad5 regimen. (**E**) Summary of

Ad5 hexon-specific antibody responses in sera. (**F**) Summary of spike-specific CD8 T cells in PBMCs. (**G**) Summary of spike-specific antibody responses in sera. (**H**) Spike-specific plasma cell responses in bone marrow. (**I**) Spike-specific memory B cell responses in spleen. Data from one experiment (n=5 mice per group). Experiment was repeated once with similar results. In panels B,C, E, and F indicated *P* values were calculated by Mann-Whitney t test (p value from panel F is from week 8). In panel G, indicated *P* value was calculated by Welch's t test. Error bars represent SEM.

Figure S4

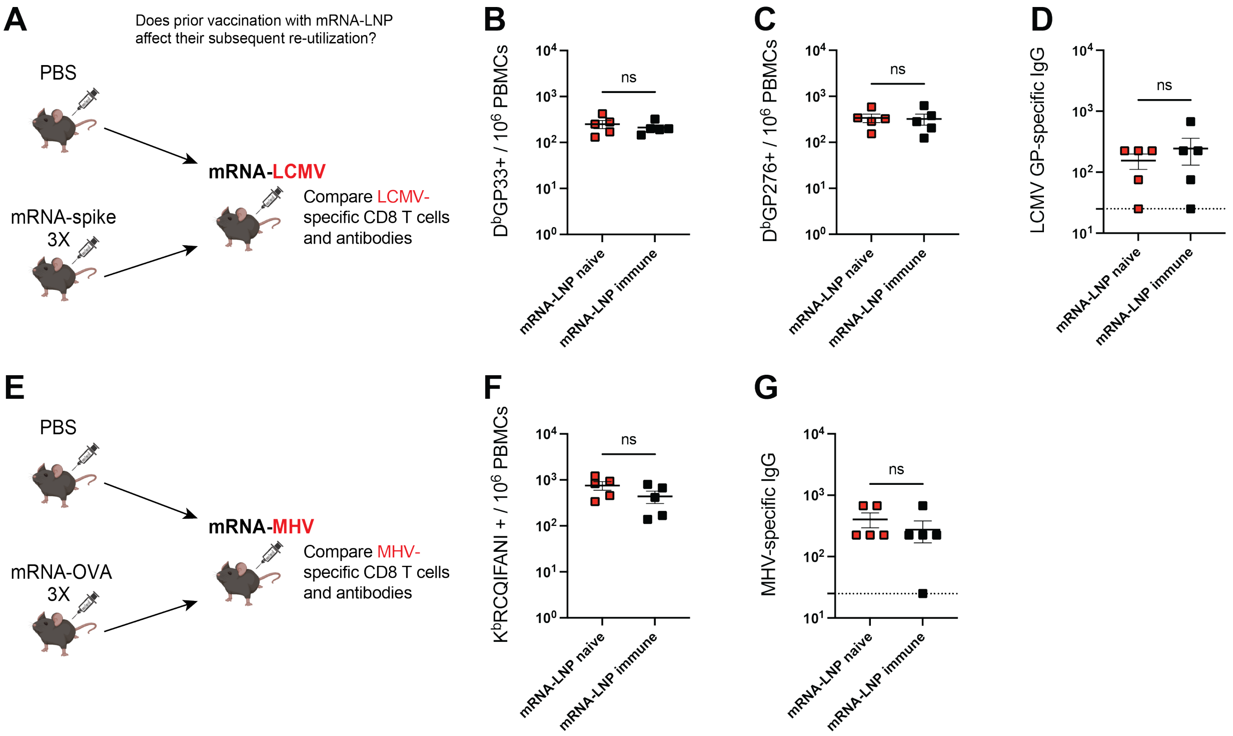

Supplemental Figure 4. Prior immunization with mRNA vaccines does not impair their subsequent re-utilization. (**A**) Experimental outline for comparing mRNA vaccine-elicited responses in mice that had been previously immunized with mRNA-spike vaccines. (**B**) Summary of LCMV GP33-specific $CD8^+$ T cell responses in PBMCs. (**C**) Summary of LCMV GP276-specific $CD8^+$ T cell responses in PBMCs. (**D**) Summary of LCMV GP-specific antibody responses in sera. (**E**) Experimental outline for comparing mRNA vaccine-elicited responses in mice that had been previously immunized with mRNA-OVA vaccines. (**F**) Summary of MHV-specific $CD8^+$ T cell responses (K^bRCQIFANI+) in PBMCs. (**G**) Summary of MHV-specific antibody responses in sera. Data from panels B, C, D, F, and G are from week 2 post-immunization. Data from one experiment (n=5 mice per group). Indicated *P* values were determined by Mann-Whitney t test. Error bars represent SEM.

Figure S5

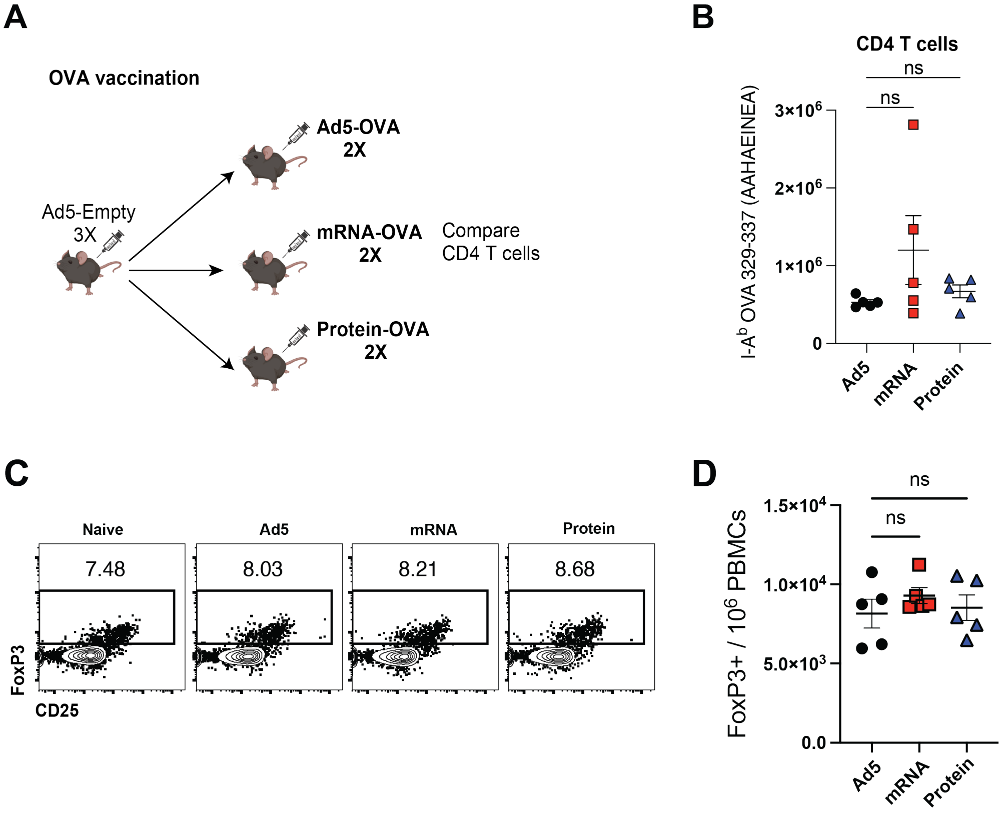

Supplemental Figure 5. Similar CD4 T cell responses among different vaccine platforms. (**A**) Experimental outline similar to Fig. 7A. (**B**) Summary of OVA-II–specific (I-A^{b} AAHAEINEA+) CD4^{+} T cells in the spleen at week 8. (**C**) Representative FACS plots showing FoxP3+ CD4+ T regulatory cells in PBMCs at week 2. (**D**) Summary of Treg responses in PBMCs at week 2. Data from one experiment (n=5 mice per group). Experiment was repeated once with similar results. Indicated *P* values were calculated by ordinary one-way ANOVA with Dunnett's multiple comparisons. Error bars represent SEM.
